# Bioinspired Design for Space Robots: Enhancing Exploration Capability and Intelligence

**DOI:** 10.3390/biomimetics11010030

**Published:** 2026-01-02

**Authors:** Guangming Chen, Xiang Lei, Shiwen Li, Gabriel Lodewijks, Rui Zhang, Meng Zou

**Affiliations:** 1 College of Mechanical and Electrical Engineering, Nanjing University of Aeronautics and Astronautics, Nanjing 210016, China; 2 Key Laboratory of Bionic Engineering (Ministry of Education), Jilin University, Changchun 130025, China; 3 College of Engineering, Science and Environment, University of Newcastle, Callaghan, NSW 2308, Australia

**Keywords:** biomimetic design, space robotics, granular soil, planetary exploration, bioinspired locomotion

## Abstract

Space exploration is a major global focus, advancing knowledge and exploiting new resources beyond Earth. Bioinspired design—drawing principles from nature—offers systematic pathways to increase the capability and intelligence of space robots. Prior reviews have emphasized on-orbit manipulators or lunar rovers, while a comprehensive treatment across application domains has been limited. This review synthesizes bioinspired capability and intelligence for space exploration under varied environmental constraints. We highlight four domains: adhesion and grasping for on-orbit servicing; terrain-adaptive mobility on granular and rocky surfaces; exploration intelligence that couples animal-like sensing with decision strategies; and design methodologies for translating biological functions into robotic implementations. Representative applications include gecko-like dry adhesives for debris capture, beetle-inspired climbers for truss operations, sand-moving quadrupeds and mole-inspired burrowers for granular regolith access, and insect flapping-wing robots for flight under Martian conditions. By linking biological analogues to quantitative performance metrics, this review highlights how bioinspired strategies can significantly improve on-orbit inspection, planetary mobility, subsurface access, and autonomous decision-making. Framed by capability and intelligence, bioinspired approaches reveal how biological analogues translate into tangible performance gains for on-orbit inspection, servicing, and long-range planetary exploration.

## 1. Introduction

Exploring the universe beyond human presence is significant for scientific knowledge and resource utilization [1]. Contemporary space exploration has entered a new era as multiple nations set unprecedentedly ambitious goals. China has expanded its space station with new modules. Both China and the United States plan to explore the extreme environment of the lunar south pole. The European Space Agency is conducting a detailed post-impact survey of a target asteroid [2]. To meet these objectives, diverse space robots are being developed [3,4].

Nonetheless, current robots face significant challenges due to the constraints of the space environment. Wheeled robots, for example, often experience sinkage and limited adaptability on lunar and planetary surfaces. Current systems also suffer from limited mobility on steep or granular slopes, insufficient anchoring capability on vertical or irregular structures, degraded sensing performance under dust and extreme lighting, reduced reliability under radiation and thermal cycling, and constrained autonomy due to communication delays and computational limits. These limitations directly hinder mission safety, operational precision, and surface-access capability. Consequently, innovative systems with improved capability and intelligence are required [5,6].

Animals navigate complex environments efficiently, and their morphology and behaviors can inspire robotic design [7]. “Bioinspired design” describes the use of biological principles—structural, behavioral, or control-related—to enhance robotic performance and robustness in space. As a result, bioinspired strategies enhance adhesion and manipulation, terrain mobility, and exploration intelligence. “Capability” refers to a robot’s physical performance under space environment constraints, such as adhesion, mobility, and load handling. “Intelligence” denotes a robot’s ability to perceive, plan, adapt, and make decisions under uncertainty and communication delay. Applications extend from space debris capture and space station inspection [8] to lunar or planetary surface construction [9] and asteroid or lava cave exploration [10,11]. To improve mission success under harsh conditions, it is essential to investigate bioinspired solutions tailored to space environments. 

Existing reviews cover rigid robotic arms for on-orbit servicing [12], bioinspired surface robots for lunar terrain [13], attachment mechanisms for extraterrestrial applications [14], and compliant/soft robotics for space missions [15]. However, recent advances—such as biomimetic flapping robots for Mars—remain under-discussed [16], and bioinspired algorithms and control for space missions have not yet been comprehensively analyzed. Moreover, the general methodology for transferring biological functions to space robots has not been proposed.

This review is based on a literature survey of the recent ten-year (2016–2025) research on bioinspired robotics for space exploration. In contrast to prior work, it provides an integrated, cross-domain synthesis that unifies adhesion, manipulation, terrain mobility, and exploration intelligence under a single biologically grounded framework. It uniquely emphasizes quantitative performance metrics, environment-dependent constraints, and the end-to-end translation of biological principles into robotic mechanisms, controllers, and mission capabilities. Therefore, this paper offers a more holistic perspective and identifies cross-cutting design principles that have been overlooked.

## 2. Adhesion and Manipulation

On-orbit services are essential to safe, long-duration space station operations, including debris capture, inspection, and in-space assembly. To assist astronauts in conducting these tasks, bioinspired adhesive and manipulation devices based on biological grasping and manipulating features have been developed. 

### 2.1. Adhesive Devices

Biological systems, such as geckos, rely on hierarchical fibrillar structures—composed of lamellae, setae, and spatulae—that generate adhesion via van der Waals forces. These tiny structures naturally self-align, maximizing surface conformity even on micro-roughness. Inspired by this, space-grade synthetic adhesives now incorporate microfibril arrays, anisotropic surface patterns, and compliance gradients to reproduce gecko-like attachment with minimal preload. Such bioinspired microstructures also enable rapid detachment through controlled peeling, making them highly suitable for microgravity, where inertial forces are low. For attachment, the grippers extend to form a tight contact with the surface due to its high stiffness, and the adhesion force is relatively strong. When the grippers bend, the contact surfaces become less due to the contraction of the foot structure, enabling a clean and residue-free detachment. 

**Figure 1:**
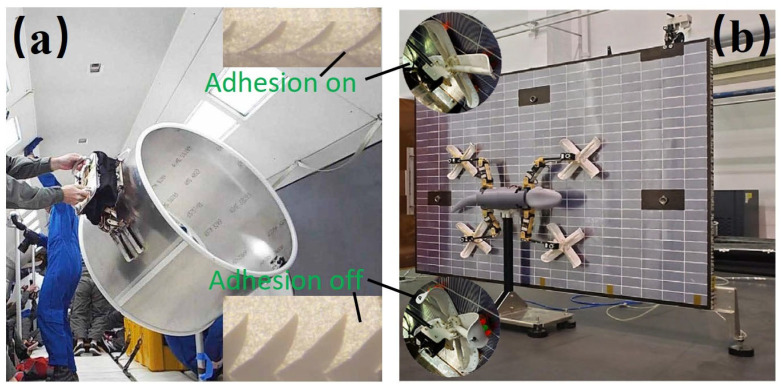
Bioinspired adhesion devices. (**a**) Gecko-toe-inspired gripper [18]. Adapted with permission from Ref. [18]. Copyright 2017, Jiang et al. (**b**) Gecko-inspired robot climbing on a solar panel [19]. Adapted with permission from Ref. [19]. Copyright 2024, Wiley-VCH GmbH.

To grasp objects with varied sizes and shapes, space robotic manipulators have been conceptualized [17]. Gecko-toe-inspired dry adhesives enable adherence on large, smooth spacecraft surfaces in vacuum, with almost no squeeze force and almost no power, and release cleanly on command. One type of gecko-inspired adhesive gripper (manufactured from Stanford University, Stanford, USA), for grasping large objects is shown in Figure 1a. The pad sizes range from 3 to 8 cm, demonstrating shear adhesion of 20–30 N/cm^2^ and normal adhesion of 5–10 N/cm^2^. It assists astronauts in capturing and manipulating large objects in microgravity [18] (Figure 1a). This adhesive ability is also effective when tested on the International Space Station. Likewise, a gecko-inspired robot (fabricated from Nanjing University of Aeronautics and Astronautics, Nanjing, China) with variable-stiffness adhesive toes can climb at speeds of around 1.8–2.5 cm/s on smooth or rough surfaces, enabling inspection and solar panel monitoring for space structures (Figure 1b) [19]. To enhance durability under lunar or Martian regolith, self-healing polymer coatings can repair micro-abrasions that degrade adhesion, while nanostructured dust-repellent layers minimize particle buildup. Together, these treatments help preserve effective contact area and reduce adhesion loss.

### 2.2. Grasping and Handling Devices

Elephants exhibit exceptional dexterity through their muscular hydrostat trunks, which allow omni-directional bending, twisting, elongation, and fine motor control without any bones. This principle is replicated in continuum robots using fiber-reinforced elastomer actuators and tunable-stiffness spines. Similarly, crab claws possess dual-function gripping—fine pinching and broad clamping—driven by exoskeletal leverage systems. Robotic end-effectors now adopt analogous passive-locking joints and friction pads that stabilize objects of irregular geometry. In beetles, force amplification arises from the mechanical advantage of multi-segment legs and the deformation of the exoskeleton, which stores elastic energy and releases it rapidly during lifting or gripping. The beetle-inspired robot leverages these passive mechanical advantages to generate higher grip forces and support payloads 2–3 times its own mass while keeping power consumption low. 

**Figure 2:**
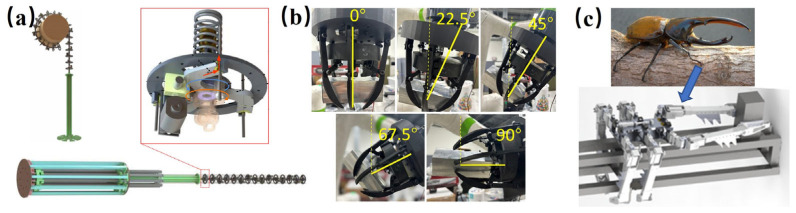
Bioinspired grasping service. (**a**) Elephant-trunk-inspired continuum robot [20]. Reprinted with permission from Ref. [20]. Copyright 2025, IEEE. (**b**) Crab-claw-inspired end effector [21]. Adapted with permission from Ref. [21]. Copyright 2025, Elsevier Inc. (**c**) A real Beetle and biomimetic robot for handling on a space truss [23]. Reprinted with permission from Ref. [23]. Copyright 2024, Shi et al.

Inspired by the elephant’s trunk, a stiffness-controllable continuum robot (manufactured from Southeast University, Nanjing, China) enables gentle yet firm grasping of large, irregular debris [20] (Figure 2a). Its payload manipulation ranges from 2 to 5 kg, with 180–220° bending flexibility. The device can be soft for safe capture and then stiffened for accurate transport. A crab-claw-inspired multifunctional end-effector (manufactured from Purdue University, West Lafayette, USA) can pinch irregular hardware with a 10–25 N force and 75–90% scooping efficiency from various perspectives. This supports effective grasping, assembly, and in-space manufacturing [21] (Figure 2b). In parallel, beetle-inspired systems give legged robots a simple, power-lean way to handle cargoes on space trusses with high stability and low risk [22,23]. Figure 2c illustrates a handling robot (fabricated by Harbin Institute of Technology, Harbin, China). mimicking the Dynastes Hercules beetle with typical body lengths of 30–50 cm and a total mass of 5–7 kg. It achieves transport of payloads 2–3 times body mass along truss structures.

Figure 1 and Figure 2 show the mechanisms for gecko pads, trunk gripper, crab claw, and beetle feet. These map to shear/normal adhesion, load/area, peel force, cycles, tip/slip force, diameter range, anchor force, node-transition success, and payload fraction. Table 1 compiles these metrics with their test environments.

## 3. Terrain Mobility

To operate on the Moon and Mars, robots must traverse granular slope regolith, rough, and rocky terrains. Exploration of asteroids also requires climbing robots that are adaptive to various steep cliffs. This section reviews granular-moving and digging systems, rock and cliff climbers, and jumping and flying robot concepts.

### 3.1. Granular Regolith Adaptability

Animals such as desert lizards and sandfish leverage body undulation, limb phasing, and substrate-engaging claws to move efficiently through loose sediments. These creatures adjust limb penetration depth and body curvature based on ground compliance. Translating these principles, bioinspired planetary robots now use flexible spines, wide contact feet, and adjustable gait patterns to minimize slip, reduce sinkage, and increase thrust on granular regolith. Mole crab- and mole-inspired burrowers employ oscillatory digging, asymmetric tooth-like cutting surfaces, and body anchoring segments—biological strategies that increase penetration efficiency and reduce excavation energy in compacted soils.

Quantitatively, terrain adaptability can be assessed through the slip ratio, where maintaining less than 10–15% slip indicates stable thrust in low gravity. Soil-compaction resistance also reflects locomotion effectiveness, as a 20–30% reduction in required penetration force signifies more efficient engagement with loose regolith. Specific energy consumption (J/m/kg) further captures adaptability, with minimized slip and sinkage typically lowering the cost of transport by 10–20%.

**Figure 3:**
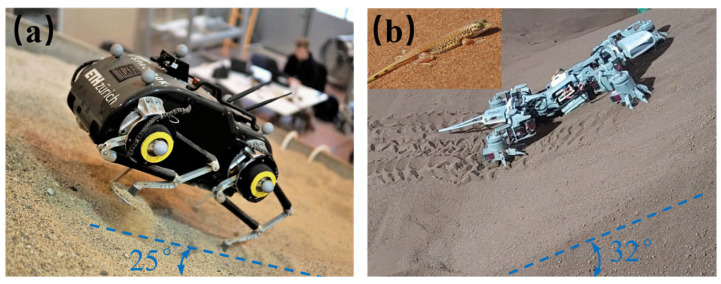
Bioinspired quadrupeds on granular terrain. (**a**) SpaceBok climbing a slope [24]. Reprinted with permission from Ref. [24]. Copyright 2025, Bapat et al. (**b**) Desert-lizard-inspired robot on soft slope ) [26].

To improve locomotion on loose terrain and slopes, bioinspired quadrupeds with special foot structures have been developed. The SpaceBok robot (fabricated from Robotic Systems Lab, ETH Zurich, Switzerland), measuring 60–70 cm in height and weighing 20–25 kg, with rounded feet and spines, climbs steep Martian analogue slopes around 25° (Figure 3a) [24]. It achieves a 0.1–0.3 m/s walking speed, with lunar jump heights of 1–1.5 m. Desert-lizard-inspired crawling robots (fabricated from Nanjing University of Aeronautics and Astronautics, Nanjing, China). can actively grasp soil and swing their flexible spines, which can stably traverse Martian analogous soft flat terrain at 7.1 mm/s [25]. The length, width, and height are 741 mm, 378 mm, and 105 mm, and its mass is around 4.5 kg. By alternately lifting and lowering the trunk on contact terrains, they can traverse slopes up to 32°, corresponding to the slip ratio (Figure 3b) [26].

**Figure 4:**
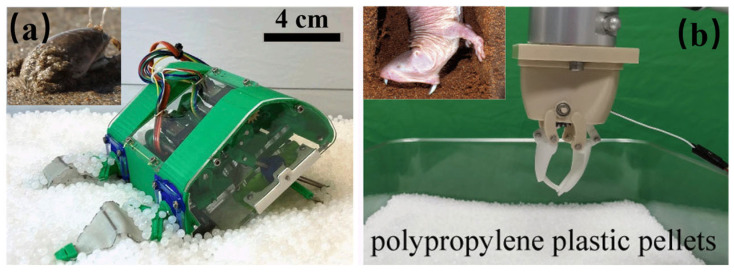
Bioinspired burrowing. (**a**) Mole crab-inspired burrowing robot [28]. Reprinted with permission from Ref. [28]. Copyright 2022, Treers et al. (**b**) Mole-incisor-inspired burrowing mechanism [29]. Adapted with permission from Ref. [29]. Copyright 2022, IEEE.

Inspired by sand-digging animals, burrowing robots enable movement on and beneath lunar or Martian regolith [27]. A mole crab-inspired legged burrowing robot (manufactured from University of California, Berkeley, USA), is shown in Figure 4a [28], having a body length, width, and height of 10, 4.8, and 9.8 cm and a mass of 310 g. Because its oscillatory leg motions and scoop-shaped appendages fluidize and locally loosen the surrounding regolith, lowering effective soil compaction, it can achieve a downward penetration speed of 2–5 cm/min, with 30–50% resistance reduction. A burrowing mechanism inspired by mole incisors (manufactured from Guangdong University of Technology, Guangzhou, China), shown in Figure 4b, is naturally suited for fracturing compact, arid soils analogous to dense regolith of the planetary subsurface. It has dimensions of 40–45 cm in length and 1–2 kg in mass. The maximum output torque is 448 N cm. It achieves forward progress rates of 1–3 cm/min in dense simulants and penetration force reduction by 20–35% compared to conventional drills [29]. When a mole incisor is paired with high-torque, low-power actuators operating at slow, energy-efficient speeds, the system minimizes wasted work against dense regolith. Together, these mechanisms lower excavation energy while improving depth gained per unit power.

### 3.2. Rock and Cliff Climbing

Climbing organisms, such as insects, goats, and beetles, employ specialized attachment strategies—spiny microhooks, hoof-like compliant pads, and distributed limb loading—to maintain stability on steep and irregular surfaces. Biological microspine mechanics are translated into robotic reality by using sharp, hardened spine tips and compliant flexures that allow multiple spines to share load and engage rock asperities reliably. This ensures that even on uneven surfaces, some spines always achieve secure purchase, maintaining stability during weight transfer. As a result, these load-sharing, passively compliant spine arrays enable consistently high node transition success rates. Robots inspired by these climbers now integrate passive microspines, adaptive wrist joints, underactuated hoof-shaped grippers, and body weight distribution strategies that mimic natural climbing biomechanics.

**Figure 5:**
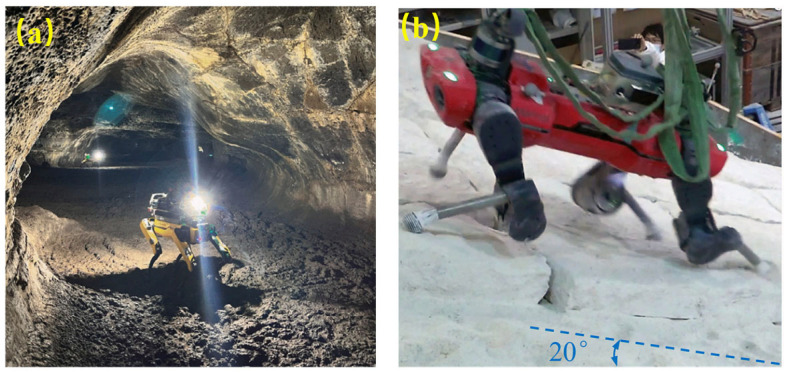
Bioinspired robots for lava cave exploration. (**a**) Quadruped tested in a lava cave analogue [30]. Adapted with permission from Ref. [30]. Copyright 2024, Elsevier Inc. (**b**) ANYmal on granular and rocky surface [9]. Adapted with permission from Ref. [9]. Copyright 2023, IEEE.

To explore the rocky areas on the surface of Mars or lava cave grounds, the quadruped spot developed by Boston Dynamics company (Cambridge, MA, USA) and Jet Propulsion Laboratory (California Institute of Technology, Pasadena, CA, USA), has been tested in terrestrial lava cave analogues. This robot has a standing height of ~0.84 m, a body length of ~1.1 m, and a mass of 30–32 kg. The results demonstrated that this robot could navigate uneven floors, ledges, and tight corridors in real lava caves and cave-like environments (Figure 5a) [30]. Its maximum walking speeds are up to 1.6 m/s while maintaining slip rates less than 10%. The knee-crawling ANYmal robot (manufactured from ETH Zurich, Zurich, Switzerland) possesses a standing height of 0.6–0.7 m, a body length of 0.8–1.0 m, and a mass of 30–50 kg, depending on its configuration. When trained with a stability margin objective, it extends walking speeds up to 1.5 m/s, traversing abilities >20°, and slip rates <10% on rock slabs dusted with sand (Figure 5b) [9,31].

**Figure 6:**
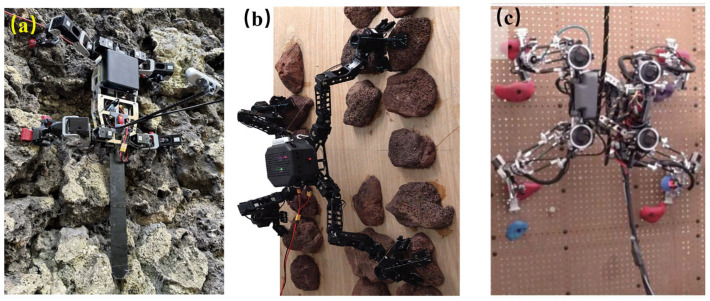
Bioinspired cliff climbing. (**a**) LORIS free-climbing robot [33]. Reprinted with permission from Ref. [33]. Copyright 2024, IEEE. (**b**) MARCbot with spiny bioinspired feet [34]. Reprinted with permission from Ref. [34]. Copyright 2024, Wiley-VCH GmbH. (**c**) SCALER robot with goat-hoof-inspired feet [35]. Reprinted with permission from Ref. [35]. Copyright 2025, IEEE.

For near-vertical walls and asteroid surfaces, specific bioinspired feet have been integrated into legged robots [32]. LORIS (manufactured from, Carnegie Mellon University, Pittsburgh, PA, USA) employs passive spine grippers on a passive three-degree of freedom wrist, supporting 20–40 N hold force per limb, and can vertically climb on cinder-block walls and uneven rock at speeds of 0.3–0.5 cm/s (Figure 6a) [33]. The overall body length is 30–40 cm, and the total mass is 3.2 kg. A multimodal rock-climber MARCbot (manufacture from Beihang University, Beijing, China), using semi-passive spine grippers inspired by beetles, can climb vertical rocky surfaces with 0.5–1.5 mm spine penetration (Figure 6b) [34]. The body length of MARCbot is 20 cm, with a leg extension of 39 cm, and its mass is 4.8 kg. SCALER (fabricated from University of California, Los Angeles, CA, USA) employs underactuated two-finger hoofed grippers that mechanically conform to surfaces, sustaining hold forces of 30–50 N per limb, enabling vertical, overhanging, and inverted climbing on rough surfaces (Figure 6c) [35]. Its body length is 45–60 cm, and its mass is 6.3 kg.

### 3.3. Jumping and Flying

Jumping animals—from fleas to locusts—store elastic energy in tendons or resilin pads and release it explosively for high takeoff velocity. Robotic jumpers adopt similar strategies with spring-loaded linkages, parallel elastic actuators, and energy-storage latches, enabling traversal of obstacles much larger than their body heights. Insects capable of flight in low-density air maintain lift through high wingbeat frequencies, large stroke amplitudes, and flexible wing membranes. Flapping-wing robots translate these features by integrating lightweight transmission systems, compliant wing spars, and aerodynamic scaling to match Martian atmospheric density.

**Figure 7:**
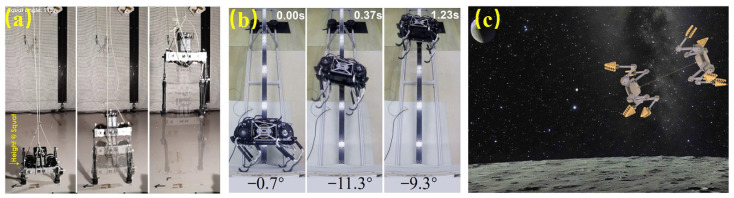
Bioinspired jumping robot mobility. (**a**) Jumping-leg prototype for cave traversal [37]. Reprinted with permission from Ref. [37]. Copyright 2024, IEEE. (**b**) SpaceBok quadruped with jumping ability [38]. Reprinted with permission from Ref. [38]. Copyright 2024, IEEE. (**c**) SPLIT-TER tethered dual-robot system [39]. Reprinted with permission from Ref. [39]. Copyright 2025, IEEE.

To overcome obstacles (e.g., crater rims, boulders), jumping or hopping robots on top of legged animals are proposed [36]. A jumping-legged prototype (manufactured from the Norwegian University of Science and Technology, Norway) was designed with a five-bar spring-assisted mechanism in each leg, can adapt to both bipedal and quadrupedal systems. Testing of the bipedal prototype demonstrated jumping 3.63 m in simulated Mars’ gravity (Figure 7a) [37], achieving takeoff velocities of 2.0–2.5 m/s, with landing impact forces reduced by 20–30% due to elastic energy recovery. A typical body height is 50–70 cm, and a typical mass is 10–15 kg. A jumping version of SpaceBok (fabricated from ETH Zurich, Switzerland) has parallel elastic legs and arc feet, with a robot height of 60–70 cm and a mass of 20–25 kg. It exhibits a powerful jump up to three times its body size, takeoff velocities of 1.5–2.2 m/s, and horizontal hop distances of 2–3 m in simulated lunar gravity (Figure 7b) [38]. A tethered dual-robot system SPLITTER (fabricated from University of California, Los Angeles, CA, USA), with a module length of 30–40 cm and a mass of 4–6 kg, is shown in Figure 7c. It achieves coordinated leap distances of 1–2 m in simulated lunar gravity, maintaining tether tension control within ±5% and enabling shared stabilization that reduces tip-over risk by 30–40% [39].

Scaling laws from animal jumping show that jump height depends on maximizing elastic energy relative to body weight, so optimizing the five-bar legs focuses on tuning spring stiffness and preload to store more energy efficiently. Matching dimensionless parameters from high-performance jumpers—such as normalized power and tendon stiffness—helps concentrate actuator work into a short loading phase. This allows elastic elements to release energy rapidly at takeoff, boosting jump height with minimal power.

**Figure 8:**
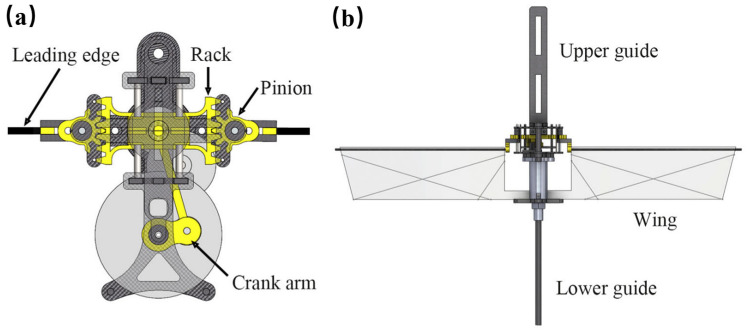
Flapping-wing robot for adapting to Martian conditions: (**a**) mechanical transmission structure; (**b**) flapping-wing structure [41]. Reprinted with permission from Ref. [41]. Copyright 2025, Lim et al.

The simulated Martian environment demonstrates dynamic similarity with insects on Earth and is achieved by preserving the relevant dimensionless parameters. For application in the Mars surface’s low, thin atmosphere, the wings of insects are scaled three to four times their normal size [40]. Figure 8a,b illustrates the insect-inspired KUBeetle ( fabricated from Konkuk University, Seoul, Republic of Korea), adopting a rack-pinion flapper with a wing length of 9 cm and a total mass of 11.7 g. Using 25–35 cm wingspans and 35–40 Hz wingbeat frequencies, it promotes sufficient lift coefficients for sustained hovering at 0.6% Earth atmospheric density and demonstrates stable attitude control with less than 5° oscillation during hover tests. These results indicate that flapping robots are applicable for low-density flight on Mars [41]. By scaling wings 3–4× larger, flapping robots raise wing area and chord enough to partly offset Mars’ ∼50–100× lower air density, preserving Reynolds number similarity when combined with comparable tip velocities. This also restores thrust-to-weight balance by generating sufficient lift for hovering and attitude control in a thin CO_2_ atmosphere.

Figure 3, Figure 4, Figure 5, Figure 6, Figure 7 and Figure 8 illustrated granular quadrupeds, burrowing mechanisms, cliff climbers, and jumping systems. Their capabilities are evaluated in terms of slope, slip ratio, sinkage, anchor/hold force and overhang ability, step/ledge clearance and recovery, and jump range/accuracy. Table 2 pairs each metric with gravity level, simulant type, temperature, and dust exposure for a fair comparison.

## 4. Exploration Intelligence

Biological organisms achieve adaptive learning, robust decision-making using distributed sensing, and decentralized coordination. This section illustrates strategies for path planning, swarm cooperation, and effective learning. These approaches boost reliability and reach by enabling fast, low-impulse planning, scalable teamwork with minimal communications, and adaptation to changing terrain and contact conditions. 

### 4.1. Adaptive Learning

Many species react to uncertain and dynamic environments with rapid, low-energy behavioral adjustments—features highly desirable in space robotics. Bioinspired intelligence thus seeks to replicate animal-like perception through interaction-driven learning to improve autonomy under harsh space conditions [42,43].

**Figure 9:**
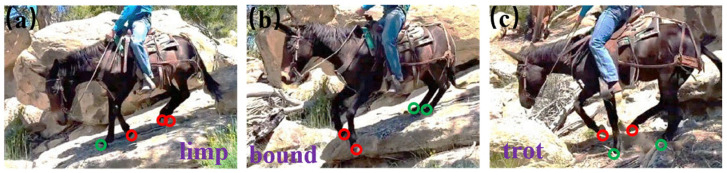
Auxiliary gaits for challenging terrain, in which red and green circles respectively indicate swing and stance feet: (**a**) limb sequence; (**b**) bound; (**c**) trot [44]. Reprinted with permission from Ref. [44]. Copyright 2025, Humphreys et al.

Interaction-driven learning is often quantified through adaptive force modulation. Figure 9a–c illustrate how a horse adjusts auxiliary gaits to traverse complex terrain in accordance with mechanical interactions. It adjusts limb stiffness by 20–40% within a few strides after encountering uneven ground. These adjustments emerge from trial-to-trial error correction, where sensory feedback (e.g., load, slip, vibration) produces measurable neural gain updates of 5–15% per interaction cycle. In robotic systems, these principles are implemented through online adaptation modules that update impedance, foothold selection, or gait phase using interaction forces; typical implementations achieve 15–30% reductions in slip rate and 20–50% faster recovery from disturbances, and they can converge on stable gaits within 5–20 interaction episodes even on highly irregular terrain. These gaits expand the feasible motion and improve robustness in overcoming nasty terrains [44].

### 4.2. Path Planning and Cooperation 

In biological systems, path-planning often emerges from local interactions among neurons or individuals—for example, ant foraging networks or neural field activation patterns. These principles inspire computational models that favor robustness and distributed decision-making rather than centralized control. 

**Figure 10:**
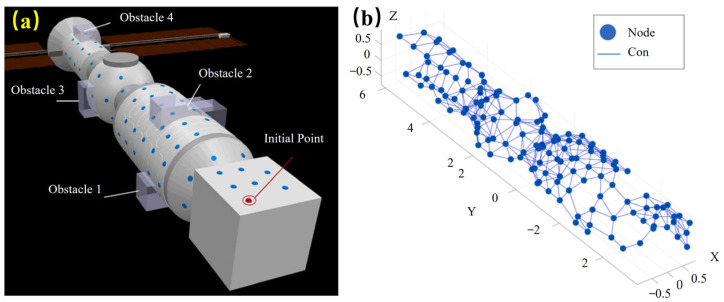
Path planning on a space structure: (**a**) full coverage on a station surface; (**b**) GBNN network corresponding to grasp fixtures [45]. Reprinted with permission from Ref. [45]. Copyright 2025, Elsevier Inc.

Bioinspired path planning has improved manipulation and locomotion in on-orbit and surface operations. A path-planning strategy based on an improved Glasius Bioinspired Neural Network (GBNN) discretizes the workspace into 50 × 50–100 × 100 neural nodes, with each node updated by local diffusion and inhibition terms using time steps on the order of 1–5 ms. The GBNN reflects biological path-planning by using local, low-energy node updates that yield efficient paths with low computational cost. It achieves planning latencies of 20–40 ms per iteration, obstacle-avoidance success rates above 95% in cluttered mock station environments, path-optimality gaps under 10% compared to global planners, and a computational load reduction of 30–50% relative to classical neural-field methods [45] (Figure 10). Its fast per-step latency resembles distributed neural processing, and its ability to reshape activation patterns under new constraints mirrors animal-like adaptability to uncertainty.

Bioinspired swarm strategies increase the efficiency in conducting exploration missions. For lunar ice extraction, leafcutter ant-inspired labor division is implemented using response threshold models, where each robot is assigned a numerical activation threshold and task utility updates dynamically modify recruitment probabilities by 20–40% (Figure 10a). Firefly-inspired communication is constructed using phase-oscillator controllers, where each robot broadcasts low-bandwidth light or radio pulses and adjusts its internal clock by a coupling gain (0.1–0.3) (Figure 1b) [46]. Lunarminer mirrors this with special robots, which can reduce tasking time by up to 40% and save energy by 31% in Shackleton-style simulations.

**Figure 11:**
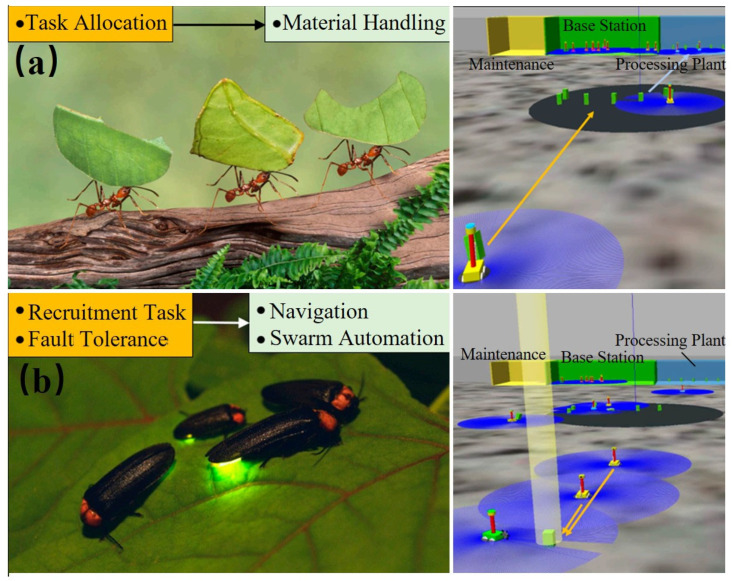
Nature-inspired swarm concepts for lunar ice mining, in which the blue-shaded areas illustrate the zones covered by each unit and the arrows represent transport paths: (**a**) division of labor (leafcutter ants); (**b**) light-signal-inspired communication (fireflies) [46]. Adapted with permission from Ref. [46]. Copyright 2024, Tan.

Figure 9, Figure 10 and Figure 11 depict robot intelligence based on neural planners, swarms, and learning via interaction. This intelligence is evaluated in terms of planning latency, success rate, optimality gap, safety violations, map completeness, sample efficiency, regret, recovery, coverage, and robustness (N–1). Table 3 lists these alongside stressors and compute/power limits.

## 5. Bioinspired Design Methodology

The previous sections illustrate bioinspired designs using biological structures, behaviors, and strategies. This section presents an analysis of the transfer of biological models to robotic applications. Moreover, a design methodology for transferring biological functions into space robotics is outlined in accordance with the design principles.

### 5.1. Biological Analogues to Practice

Table 4 summarizes biological analogues mapped to robotic systems, highlighting the corresponding robotic designs and their capabilities/intelligence. Representative systems were validated through environmental testing and analogue trials. Reported protocols include verifications under microgravity tests and terrain trials on planetary analogue sites (e.g., granular simulants, lava caves) [47]. Air-thin/low-g flight concepts were evaluated under Mars-like pressure conditions, while cliff-climbing robots demonstrated adhesion/anchoring on rough rock and cinder block walls.

### 5.2. Design Methodology

**Figure 12:**
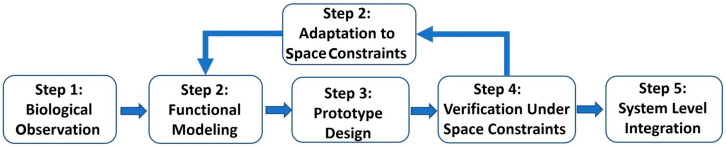
Bioinspired design scheme for space robots.

Figure 12 illustrates a key strength of the five-step bioinspired design scheme—biological observation → functional abstraction and modeling → robotic prototyping → environment-relevant testing → system-level integration. It creates an explicit traceability chain from biological function to robotic implementation.

Step 1: Biological Observation—quantitative characterization of biological performance. Designers document measurable biological attributes (e.g., gecko adhesion forces of 20–30 N/cm^2^, mole crab digging stroke patterns of 3–5 Hz). These metrics become biological baselines that anchor later engineering trade-offs.

Step 2: Functional Modeling—formalizing biological mechanisms into analyzable models under space conditions. Observed biological functions are translated into mathematical or computational models (e.g., fibrillar adhesion mechanics, response threshold models, hoof compliance curves). These models establish clear causal links between biological structure/behavior and the resulting performance [48].

Step 3: Prototype Design—mapping modeled functions onto robotic hardware or algorithms. Each robotic subsystem is explicitly derived from a biological model (e.g., microfibril geometry → synthetic adhesive pad pitch; muscle-like hydrostat bending → continuum robot stiffness profile). This ensures that every design parameter traces back to the biological principle identified in Step 1.

Step 4: Verification Under Space Constraints—testing biological hypotheses through robotic performance. Robots are evaluated in analogue environments, such as vacuum chambers, regolith bins, cliff slabs—allowing designers to quantify how well the biological function transfers. Deviations from expected biological performance (e.g., lower adhesion at −80 °C) trace back to specific model assumptions in Step 2.

Step 5: System Level Integration—embedding validated functions into multi-robot or mission architectures. By integrating multiple bioinspired modules (adhesion + climbing + swarm coordination), designers preserve a record of biological lineage: each capability is tagged with its biological origin, modeled function, tested performance, and final system role.

## 6. Comparative Analysis, Challenges, and Perspectives

Bioinspired robotics offers meaningful advantages for space exploration—adaptability, mechanical efficiency, and robustness—but deployment barriers remain. This section discusses discrepancies of bioinspired designs, core challenges, and future opportunities.

### 6.1. Comparative Analysis

(1) Limitations.

Adhesives lose performance at low temperatures; microspines underperform on fractured or dusty rock; flapping robots face mass–power–lift constraints; learning algorithms rely on hardware not yet space-qualified.

(2) Key gaps.

Key gaps include insufficient biomechanical-to-robotic modeling; scarce long-term fatigue data for soft actuators and synthetic adhesives; and minimal integration across locomotion, adhesion, sensing, and intelligence.

(3) Benchmarking Needs.

Cross-platform comparisons require standardized protocols: (i) adhesion tests −150 °C to +120 °C with dust exposure; (ii) regolith trials using standard simulants; (iii) climbing tests on substrates with known roughness/tensile strength; and (iv) intelligence benchmarks under realistic comm delays and low-power processors.

(4) Open problems.

Fail-safe adhesion/anchoring on fractured surfaces, energy-efficient hopping/flapping in low-density atmospheres, resilient swarm coordination under sparse communication, safe online learning, and integration of multiple bioinspired modules into mass- and power-limited systems remain open problems.

### 6.2. Challenges

(1) Robustness in Uncertain Environments.

Bioinspired intelligence improves adaptability, yet reliable operation under vacuum, cryogenic temperatures, dust exposure, and low-gravity dynamics remains insufficiently validated. Systems require stronger guarantees for disturbance rejection, sensing degradation, and unexpected terrain.

(2) Material Durability and Environmental Resistance.

Adhesive pads, microspines, and soft actuators degrade under dust abrasion, thermal cycling, and radiation. Long-duration endurance testing (>1000 cycles), abrasion studies, and radiation aging remain sparse, limiting lifetime prediction [49].

(3) Energy Consumption and Power Constraints.

Legged, hopping, and flapping robots often suffer a higher cost of transport than wheeled designs without optimized actuation. Energy harvesting, low-power drives, and energy-aware control remain underdeveloped [50].

(4) Multi-Robot Coordination Under Realistic Constraints.

Swarm strategies assume reliable communication and sensing, but lunar lava tube or Martian pit conditions impose delays (2–10 s) and >30% packet loss. Scalable, decentralized cooperation under such constraints remains unresolved.

### 6.3. Perspectives

(1) Robust Adaptive Intelligence.

Reinforcement learning and online adaptation techniques should be further developed to enable robots to learn from their interactions with the environment. Adaptive gaits and multimodal transformation will be key in ensuring robots can adapt in real-time [51].

(2) Durable and Self-Healing Bioinspired Materials.

To overcome durability challenges, the integration of material techniques is crucial for maintaining the longevity and reliability of bioinspired robotic systems. Gecko-inspired devices could be paired with dust-repelling coatings to ensure consistent performance on the Moon or Mars.

(3) Energy-Efficient Actuation and Power Systems.

Future developments should focus on low-power actuators, energy harvesting, and lightweight, high-capacity batteries to extend mission durations. Coupled with energy-efficient algorithms, bioinspired robots could operate autonomously for longer periods.

(4) Scalable Multi-Robot Autonomy.

By leveraging bioinspired swarm coordination and communication strategies, especially for large-scale missions, these robots can work together efficiently without a centralized controller. Future research should focus on scalable multi-robot systems that can perform cooperative tasks while maintaining autonomous decision-making under communication delays.

## 7. Conclusions

This review demonstrates the value of bioinspired designs in improving the capability and intelligence of space robots. Nature-derived strategies in adhesion, locomotion, sensing, and collective behavior provide clear functional benefits: gecko-inspired adhesives enable high-shear, low-preload grasping; beetle- and mole-inspired locomotion enhances mobility on steep, granular, or subsurface terrain; swarm mechanisms drawn from ant and firefly support scalable coordination; and bioinspired neural planners, such as GBNNs, offer efficient, real-time navigation.

Despite these advances, many bioinspired systems have not been validated under mission-relevant stressors, such as cryogenic temperatures, dust abrasion, radiation exposure, and low-gravity contact dynamics. Adhesion may weaken in extreme cold, microspines may slip on fractured rock, and soft actuators can suffer long-term fatigue. Energy constraints persist for legged, hopping, and flapping robots, while swarm and learning-based controllers lack demonstrated robustness under realistic communication delays and sensing degradation. System-level integration—combining adhesion, mobility, sensing, and autonomy—remains a key unresolved challenge.

Nevertheless, emerging advances in resilient materials, energy-efficient actuation, and adaptive intelligence point toward robust, long-duration bioinspired systems for future lunar, Martian, and deep-space missions. Prioritizing durability testing, standardized benchmarking, and integrated autonomy will be essential to transition these concepts from promising prototypes to reliable mission assets.

## Figures and Tables

**Table 1:** Metrics of bioinspired robot capabilities.

Capabilities	Evaluation Indicator	Environmental Restraints
Adhesion [18,19]	Load capacity, detach force, attach–detach cycles, rough surface adaptivity	Vacuum, rough surfaces, microgravity, radiation aging
Grasping [20,21]	Grasping precision/stability, collision safety, holding curvature/power, tip force	Irregular objects, smooth and rough surfaces
Handling [22,23]	Graspable diameter, load capacity, transport speed	Irregular objects, moving on irregular structures

**Table 2:** Metrics of bioinspired robot mobile capabilities.

Capabilities	Evaluation Indicator	Environmental Restraints
Granular regolith mobility [24,25,26]	Slip ratio, moving speed, maximum slope, endurance and reliability	Granular terrain, low gravity, soil particle sizes
Borrowing [28,29]	Digging force, energy per depth, durability, direction control	Soil hardness, regolith compaction, tool abrasion
Traversing rocky surfaces [30,31]	Obstacle clearance, moving speed, surface adaptability, powder, stability	Irregular surface, different sizes and surface asperities
Climbing cliffs [32,33,34]	Load capability, vertical, inverted traversing ability, powder	Low gravity, irregular surface
Jumping [37,38,39]	Jumping height/distance, takeoff/landing accuracy, impact loads	Low gravity, unknown terrain
Flying [40,41]	Lift force, operating time	Thin air atmosphere, low gravity, scalability

**Table 3:** Metrics of bioinspired robot intelligence.

Intelligence	Evaluation Indicator	Environmental Restraints
Learning via interaction [42,43,44].	Sample efficiency, data reliability, policy stability, domain shift robustness	Unknown irregular terrains
Bioinspired neural network (e.g., GBNN [45])	Planning latency, path success rate, coverage completeness, path smoothness	Non-uniform structure surface
Swarm strategies (e.g., ants and flies [46])	Task allocation efficiency, communication load, energy balance	Limited bandwidth, GPS-denied, time sync and clock drift

**Table 4:** Biological analogues informing space robot design.

Biological Models	Space Robotic Systems	Capabilities, Intelligence	Deployment Status
Gecko toe adhesion	Adhesive gripper	Adhesive grasping	Tested in ISS
Gecko adhesive climbing	Adhesive robotic feet	Smooth surface climbing	Specific for space
Elephant trunk	Flexible gripper	Versatile grasping	Specific for space
Crab hub and claw	Claw end effector	Grasping, assembly	Specific for space
Dynastes Hercules beetle	Beetle-inspired climber	Transportation, manipulation	Specific for space
Medium-sized dog	SpaceBok (spike foot)	Traversing soft slopes	Specific for space
Desert lizard	Lizard robot	Traversing soft slopes	Specific for space
Pacific mole crab digging	Mole crab burrowing robot	Burrowing downward	Specific for space
Mole incisors (digging)	Mole incisor drill mechanism	Efficient soil penetration	Specific for space
Medium-sized dog	ANYmal	Traversing rocky slopes	Adapted for space
Insect grasping	LORIS climber	Stable vertical climbing	Specific for space
Beetle/bird foot (spines)	MARCBot climber	Trotting on complex terrains	Specific for space
Mountain goat hooves	SCALER climber	Overhanging, upside-down	Specific for space
Medium-sized dog	SpaceBok (jumping legs)	Jumping over obstacles	Specific for space
Desert lizard jumping	SPLITTER tethered system	Exploring wider areas	Specific for space
Alpine bumblebee flight	KUBeetle flapping drone	Aerial exploration (Mars)	Adapted for space
Big-sized dog	JPL cave explorer	Lava cave navigation	Adapted for space
Animal rear legs	Jumping robot	Leaping over obstacles	Specific for space
Animal biomechanics	Optimal gait strategy	Versatility robustness	Potential for space
Decentralized ant colony	Dual-layer planning	Efficient path planning	Specific for space
Ant division, firefly connection	Swarm algorithm	Efficient resource collection	Specific for space

## Data Availability

No new data were created or analyzed in this study. Data sharing is not applicable to this article.
